# Phenology, seasonal abundance and stage-structure of spittlebug (Hemiptera: Aphrophoridae) populations in olive groves in Italy

**DOI:** 10.1038/s41598-019-54279-8

**Published:** 2019-11-27

**Authors:** Nicola Bodino, Vincenzo Cavalieri, Crescenza Dongiovanni, Elisa Plazio, Matteo Alessandro Saladini, Stefania Volani, Anna Simonetto, Giulio Fumarola, Michele Di Carolo, Francesco Porcelli, Gianni Gilioli, Domenico Bosco

**Affiliations:** 1 CNR–Istituto per la Protezione Sostenibile delle Piante, Strada delle Cacce, 73, 10135 Torino, Italy; 2 CNR–Istituto per la Protezione Sostenibile delle Piante, SS Bari, Via Amendola 122/D, 70126 Bari, Italy; 3 CRSFA–Centro di Ricerca, Sperimentazione e Formazione in Agricoltura Basile Caramia, Via Cisternino, 281, 70010 Locorotondo (Bari), Italy; 4 Dipartimento di Scienze Agrarie, Forestali e Alimentari, Università degli Studi di Torino, Largo Paolo Braccini, 2, 10095 Grugliasco, Italy; 5 Dipartimento di Medicina Molecolare e Traslazionale, Università degli Studi di Brescia, 25123 Brescia, Italy; 6 Dipartimento di Scienze Agro-Ambientali e Territoriali, Università degli Studi di Bari Aldo Moro, Via Amendola, 165/A, 70126 Bari, Italy

**Keywords:** Entomology, Agroecology

## Abstract

Spittlebugs (Hemiptera: Aphrophoridae) are the dominant xylem-sap feeders in the Mediterranean area and the only proven vectors of *Xylella fastidiosa* ST53, the causal agent of the olive dieback epidemic in Apulia, Italy. We have investigated the structured population phenology, abundance and seasonal movement between crops and wild plant species of both the nymphal and adult stages of different spittlebug species in olive groves. Field surveys were conducted during the 2016–2018 period in four olive orchards located in coastal and inland areas in the Apulia and Liguria regions in Italy. The nymphal population in the herbaceous cover was estimated using quadrat samplings. Adults were collected through sweep nets on three different vegetational components: herbaceous cover, olive canopy and wild woody plants. *Philaenus spumarius* was the most abundant species; its nymphs were collected from early March and reached a peak around mid-April, when the 4^th^ instar was prevalent. Spittlebug adults were collected from late April until late autumn. *P. spumarius* adults were abundant on the herbaceous cover and olive trees in late spring, and they then dispersed to wild woody hosts during the summer and returned to the olive groves in autumn when searching for oviposition sites in the herbaceous cover. A relatively high abundance of *P. spumarius* was observed on olive trees during summer in the Liguria Region. The present work provides a large amount of data on the life cycle of spittlebugs within an olive agroecosystem that can be used to design effective control programmes against these vectors in infected areas and to assess the risk of the establishment and spread of *X. fastidiosa* to *Xylella*-free areas.

## Introduction

The importance of spittlebugs in agriculture has greatly increased since the discovery of *Xylella fastidiosa* Wells (*Xf*) in Europe^1^, where the spread of the bacterium under cropping and non-cropping conditions has been associated with the spittlebug vector *Philaenus spumarius* L. (Hemiptera: Aphrophoridae)^2,3^. Even though all xylem-sap feeders should be regarded as potential vectors^4^, few of them are of epidemiological relevance in a specific disease system^5^. In the Americas, *Xf* epidemics have always been associated with sharpshooters (Hemiptera: Cicadellidae: Cicadellinae)^6^, whereas spittlebugs play the main if not exclusive, role in transmitting the bacterium in Europe^7,8^. The vector role of *P. spumarius*, the most common spittlebug species in Europe, has been proved in the Apulia region (Italy), where the insect has been found to transmit the *X. fastidiosa* subspecies *pauca* ST53, the causal agent of the dramatic dieback on olive trees^1,7,9^. The biological and the ecological aspects of xylem sap feeding fauna in susceptible crops are among the major knowledge gaps of *Xf* epidemics in Europe, and they are therefore currently considered research priorities for the EU, together with the description of the relative abundance of vector species^10,11^. Studies aiming to fill these gaps should consider the whole agroecosystem, not only specific crops, to obtain a comprehensive picture that allows the identification of epidemic drivers, including alternative hosts and hidden reservoirs of *X. fastidiosa* and/or its vectors^12,13^.

Before the discovery of *X. fastidiosa* in several areas of the Palaearctic, Aphrophoridae were not regarded as relevant pests in Europe, and hence the studies on these insects were mainly focused on the taxonomy and the polymorphism of the dominant species, the meadow spittlebug *P. spumarius*^14–20^. Few studies have investigated the biology and ecology of *P. spumarius* in natural habitats^21–28^. A large amount of biological information comes from studies conducted in agricultural systems in North America, where the meadow spittlebug was introduced in pre-industrial times and has been reported to cause economic damage to alfalfa and strawberry^29–33^. The available information on *P. spumarius* biology and ecology has recently been reviewed by Cornara *et al*.^2^. Other spittlebug species have basically been overlooked in the Old World, and little information on their biology and ecology is available^27,34–39^ (see the recent review by Cornara *et al*.^40^). As far as European olive agroecosystems are concerned, only a limited amount of preliminary information on the biology and ecology of spittlebugs in Apulia, in Spain, and in Greece is available^9,41–44^.

Following the epidemics of *X. fastidiosa* on perennial crops in the Mediterranean area (mainly olive and almond plants), the risk posed by the bacterium to European agriculture has increased considerably. Consequently, the information on the phenology, life history and host-plant association of spittlebugs is nowadays essential to understand the epidemiology of *X. fastidiosa* and to design appropriate vector control measures. The aim of this study has been to fill the knowledge gaps on the phenology, abundance and dynamics of spittlebug populations in olive groves in the Mediterranean basin. For this purpose, multiannual surveys were conducted in two olive groves in Southern Italy and in two olive groves in Northern Italy. The study sites are characterised by a wide range of climatic conditions within the warm-summer Mediterranean climate. The different climatic and agroecological conditions of the sampling sites allowed us to monitor different populations of *P. spumarius* and of other potential vectors of *X. fastidiosa* in olive orchards in the Mediterranean basin and to analyse their within- and between-year variations.

## Material and Methods

**Figure 1:**
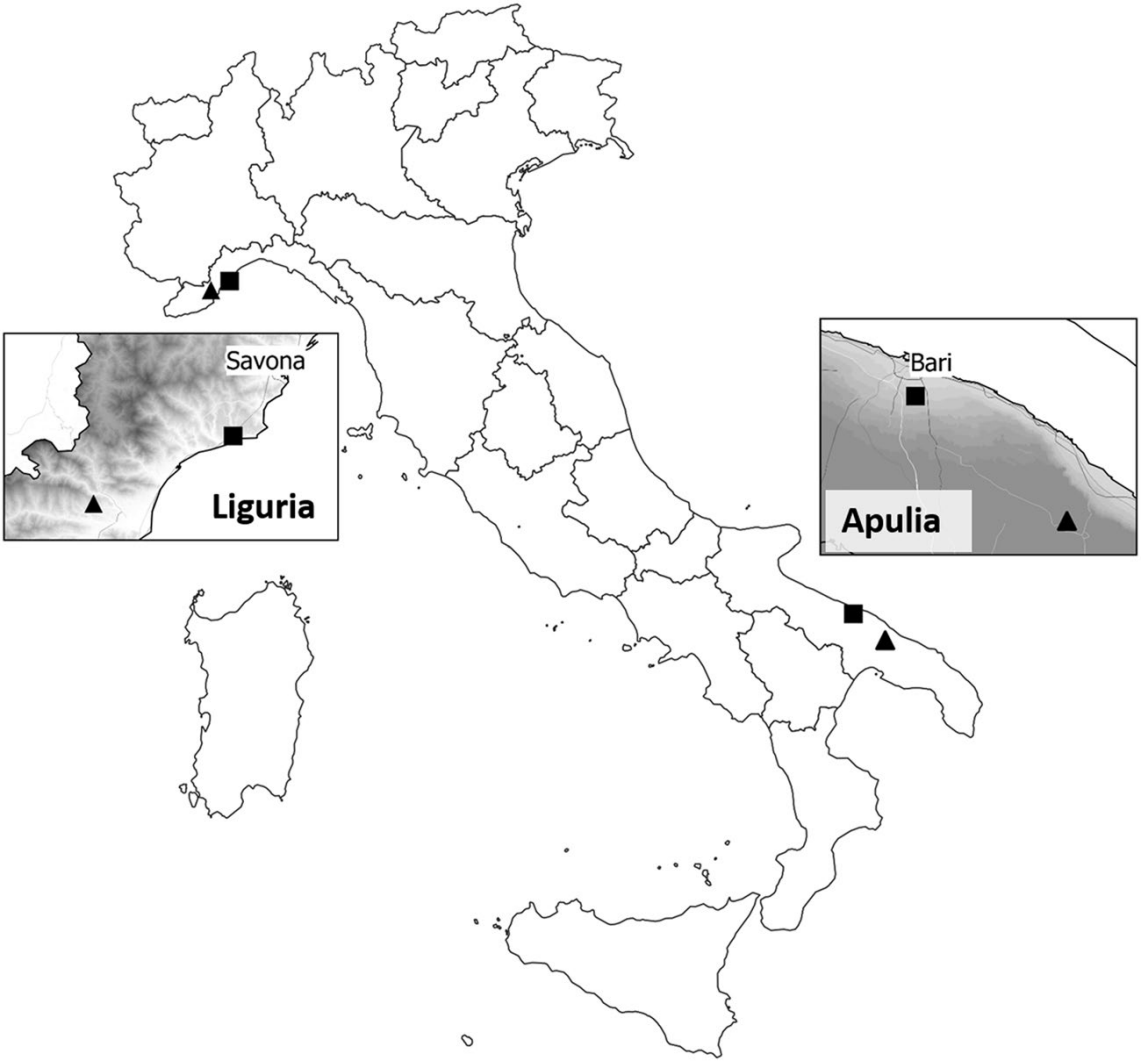
Locations of the Italian olive groves surveyed for the presence of spittlebugs during the 2016–2018 period in the Apulia and Liguria regions in Italy. The squares indicate coastal olive groves and the triangles indicate inland olive groves. The inset maps show hillshaded layer to highlight the geomorphology of the two regions.

Field surveys were carried out in olive groves in Italy during the 2016–2018 period. Four different olive groves were selected, two located in Southern Italy (Apulia – Bari Province) and two in Northern Italy (Liguria – Savona Province) (Fig. 1). The monitored olive groves in Apulia were outside the *X. fastidiosa*-infected area at the time of the survey. Olive groves were selected on the basis of a low-input management, e.g. no insecticides were sprayed and no tillage was applied in the olive groves during the study period in Northern Italy, while the soil had been ploughed in Southern Italy in June/July (thus not affecting the development of spittlebug nymphs) to avoid fire hazards during summer. One of the selected olive groves in each region was located in a coastal area and the other one in an inland area, and this choice was made in order to investigate spittlebug populations under different climatic conditions and agroecosystems within the Mediterranean basin. The sites in Apulia and Liguria are about 1,000 km apart and fall within the warm-summer Mediterranean climate area^45^. In 2017, soil tilling and the sowing of broad bean were carried out in the inland olive grove (Locorotondo municipality), making it no longer suitable for monitoring. For this reason, a new site, about 5 km from the previous one, was identified and monitored in 2017. Surveys in the Apulian olive groves only were thus prolonged to 2018. The principal characteristics of the olive groves surveys and their agroecosystems are reported in Supplementary Table S1.

A homogeneous area of 1 ha (primary sampling unit - PSU) was surveyed inside each olive grove. The nymphal populations of Aphrophoridae were monitored using the quadrat sampling method, which is frequently used to quantify the density of spittlebug nymphs^22,38,44^. At each sampling date, 30 quadrats of 0.25 m^2^ (secondary sampling unit of nymphs - SSU_n_;100 × 25 cm each) were randomly positioned on the ground cover inside each PSU. The vegetation and soil surface inside the quadrat were inspected carefully for spittlebug nymphs, which were counted, and their instars were determined directly in the field using the identification key of Vilbaste^46^. The spittlebug samplings were conservative, and the nymphs were therefore not collected in order to avoid interference with the population dynamics throughout the season. Only a few nymphs were reared to adults in the laboratory to confirm their species. The sampling of the nymphal stages was carried out weekly from early March until late-May, or until no nymphs were found in any SSU on two consecutive sampling dates. The field samplings in Apulia in 2016 were started in late March, because of unfavourable weather conditions, thus the first phases of nymphal development were not observed. 2^nd^–3^rd^ and 4^th^–5^th^ instars were aggregated because of the initial difficulties in the instar identification under field conditions.

Spittlebug adults were sampled inside the PSU of each olive grove using a sweep net (38 cm in diameter) on three different types of vegetation: herbaceous cover, olive trees and wild woody plants (shrubs or trees). The sweep net has proven to be the most reliable sampling method for spittlebug adults^43,47^. Cicadas (Hemiptera: Cicadoidea), an important group of xylem-sap feeders in Mediterranean olive groves, were not sampled because they require different sampling methods and their role in *Xf* transmission has never been confirmed, although it has been suggested in a few studies^48,49^. Samplings were carried out at randomly selected points on each sampling date to avoid repeated disturbance of the same points throughout the season. The spittlebugs in the ground vegetation were sampled in 30 randomly distributed secondary sampling units (SSU_h_). The sampling consisted of 4 sweeps performed along a 2.8-meter transect (four 0.7 m steps each). Therefore, the area effectively sampled by means of sweeping in an SSU_h_ was estimated to be about 1.0 m^2^ (0.7 m long × 0.38 m wide × 4 sweeps). One hundred and twenty sweeps were performed in the ground vegetation per site per sampling date. The insects in the olive canopies were sampled using a sweep net with a 2-meter long stick on 20 randomly distributed olive trees (an olive tree is an SSU_o_). Ten sweeps were performed on each olive tree around the entire canopy. Sampling on wild woody plants was carried out with the same methodology as that used for the olive trees, but on 10 different, randomly chosen plants (a plant corresponds to an SSU_s_). Owing to the presence of dense Mediterranean shrubland (garrigue), it was not always possible to perform the sweeps all around a single plant. The wild woody plant species present in each site were identified at a species level^50^. Sampling of spittlebug adults was carried out from the appearance of the first early adults in the quadrat samplings until October/November, or when no spittlebugs were found in any SSU on two consecutive dates. The adult spittlebug samplings were conservative, thus the collected insects were released immediately in the field after being identified. Any *P. spumarius* individuals were also sexed to determine the sex ratio trend during the season.

The air temperature and relative humidity were monitored in each olive grove using data loggers (HOBO U23-002; Onset Computer, Bourne, MA, USA). The data loggers were installed in the centre of the PSU, close to an olive trunk, 40 cm above the soil level, and they were covered by a solar radiation shield. Such a location permitted us to obtain similar weather data to those experienced by the nymphs and adults present on the ground cover. Whenever the data loggers failed to record weather data, due to technical issues, the information gaps were filled with data from the closest weather station. In order to study the phenology of *P. spumarius*, hourly air temperature data, expressed in °C, were used to calculate the degree-days (DD) according to the following formula:

$$DD=\mathop{\sum }\limits_{i=1}^{n}\frac{{\rm{\max }}[0,({T}_{i}-{T}_{0})]}{24}$$

where *n* is the number of hours in one year, from 1^st^ January to 31^st^ December; *T*_*i*_ is the air temperature at hour *i* and *T*_0_ represents the lower developmental threshold temperature for *P. spumarius*. Compared to chronological observations, phenology models, based on an accumulated heat unit (degree-days), help to describe the temperature-dependent development of insects in a more realistic way. In this study, the lowest developmental threshold temperature was set at 8 °C on the basis of the preliminary results of experiments conducted at controlled temperatures (authors’ unpublished data). Previous studies conducted by Chmiel and Wilson^32^, West and Lees^51^ and Zajac *et al*.^33^ also reported *P. spumarius* temperature thresholds well below 10 °C.

### Statistical analyses

Statistical analyses were conducted and plots were drawn up using R statistical software, version 3.4.4 (R Core Team 2017). Descriptive statistics were calculated for the nymphal and adult stages sampled during the surveys (*tidyverse* packages). The effect of the chronological time (days after first sampling), the olive grove, the sampling region, and their interactions with the adult sex ratio of *P. spumarius* were tested using GLM with a quasi-binomial error structure (due to overdispersion) (*glm* function). The best fitting model was selected using a step-wise procedure based on qAIC index (Mumln package).

## Results and Discussion

### Species composition and relative abundance

The samplings of the spittlebug populations in four olive orchards located in two distant Italian regions, Apulia and Liguria, indicated the presence of three predominant species of Aphrophoridae: *P. spumarius*, *N. campestris* and *Aphrophora alni*, the latter only being found in the Ligurian olive groves. *Philaenus spumarius* showed the highest mean densities among the spittlebugs for both the nymphal and adult stages (Tables 1 and 2)*. Philaenus spumarius* was the predominant species in both regions, contributing by 77.9% and 83.1% to all the spittlebug nymphs and by 79.4% and 73.8% to the total spittlebug adults sampled during the 2016–2018 surveys in Apulia and Liguria, respectively. The Ligurian olive groves usually showed higher densities of meadow spittlebugs than the Apulian ones. *Neophilaenus campestris* accounted for 20.6% and 14.7% of the spittlebug adults in Apulia and in Liguria, respectively. The *N. campestris* densities were usually lower than the ones registered for *P. spumarius*, with notable differences between the sampling years, olive groves, and, for the adults, between the vegetation compartments (Tables 1 and 2). *Aphrophora alni* accounted for 3.8% of the total adults in the Ligurian olive groves. Another three Cercopoidea species [*Cercopis vulnerata* (Rossi), *C. sanguinolenta* (Scopoli) (Hemiptera: Cercopidae) and *Lepyronia coleoptrata* (L.) (Hemiptera: Aphrophoridae)] were occasionally sampled as adults on the herbaceous cover in the Liguria (*C. vulnerata* and *L. coleoptrata*) and Apulia (*C. sanguinolenta*) olive groves.

**Table 1:** No. of spittlebug nymphs/sample (Mean ± SE) in the four olive groves in the Apulia and Liguria Regions of Italy.

Year	Region	olive grove	Spittlebug species								
			*Philaenus spumarius*			*Neophilaenus campestris*			*Aphrophora alni*		
2016	Apulia	coastal	0.778	±	0.099	0.111	±	0.064		0	
		inland	2.006	±	0.257	0.156	±	0.056		0	
	Liguria	coastal	4.586	±	0.428	0.23	±	0.047	0.395	±	0.088
		inland	4.573	±	0.43	0.497	±	0.082	0.472	±	0.1
2017	Apulia	coastal	1.32	±	0.14	0.32	±	0.088		0	
		inland	4.776	±	0.321	1.585	±	0.153		0	
	Liguria	coastal	7.354	±	0.523	0.856	±	0.103	0.062	±	0.019
		inland	3.155	±	0.27	1.366	±	0.15	0.1	±	0.034
2018	Apulia	coastal	0.645	±	0.12	0.318	±	0.076		0	
		inland	0.71	±	0.091	0.175	±	0.034		0	
	Liguria	coastal	—			—			—		
		inland	—			—			—

**Table 2:** No. of spittlebug adults/sample (Mean ± SE) in different vegetational compartments in the four olive groves in the Apulia and Liguria Regions.

Year	Region	Olive grove	Philaenus spumarius									Neophilaenus campestris									Aphrophora alni								
			Herbaceous SSU_h_			Olive trees SSU_o_			Shrubs/Trees SSU_s_			Herbaceous SSU_h_			Olive trees SSU_o_			Shrubs/Trees SSU_s_			Herbaceous SSU_h_			Olive trees SSU_o_			Shrubs/Trees SSU_s_		
2016	Apulia	coastal	0.139	±	0.02	0.136	±	0.02	0.103	±	0.016	0.067	±	0.015	0.04	±	0.01	0.02	±	0.007	0	±	0	0	±	0	0	±	0
		inland	0.312	±	0.04	0.22	±	0.037	—			0.02	±	0.007	0.01	±	0.006	—			0	±	0	0	±	0	—		
	Liguria	coastal	0.561	±	0.051	0.448	±	0.047	0.372	±	0.069	0.177	±	0.025	0.016	±	0.007	0.006	±	0.006	0.022	±	0.007	0.149	±	0.028	0.232	±	0.049
		inland	0.677	±	0.061	0.545	±	0.053	0.079	±	0.031	0.29	±	0.039	0.032	±	0.012	0	±	0	0.046	±	0.011	0.181	±	0.027	0.429	±	0.086
2017	Apulia	coastal	0.133	±	0.023	0.15	±	0.022	0.061	±	0.013	0.059	±	0.012	0	±	0	0.033	±	0.009	0	±	0	0	±	0	0	±	0
		inland	0.388	±	0.05	0.244	±	0.035	0.537	±	0.089	0.158	±	0.025	0.047	±	0.013	0.081	±	0.027	0	±	0	0	±	0	0	±	0
	Liguria	coastal	0.623	±	0.045	0.435	±	0.042	0.515	±	0.063	0.125	±	0.021	0.005	±	0.004	0.03	±	0.017	0.005	±	0.003	0.04	±	0.011	0.059	±	0.022
		inland	0.378	±	0.037	0.331	±	0.032	0.19	±	0.042	0.114	±	0.022	0.035	±	0.012	0.031	±	0.014	0.008	±	0.004	0.063	±	0.015	0.082	±	0.02
2018	Apulia	coastal	0.045	±	0.01	0.047	±	0.012	0.059	±	0.014	0.055	±	0.014	0.056	±	0.013	0.026	±	0.01	0	±	0	0	±	0	0	±	0
		inland	0.052	±	0.011	0.081	±	0.016	0.289	±	0.051	0.026	±	0.008	0.022	±	0.009	0.022	±	0.014	0	±	0	0	±	0	0	±	0
	Liguria	coastal	—			—			—			—			—			—			—			—			—		
		inland	—			—			—			—			—			—			—			—			—		

Note that the sample units (SSU: secondary sample unit) are different among vegetation compartments (see the material and methods section).

### Philaenus spumarius

#### Nymphal instars

**Figure 2:**
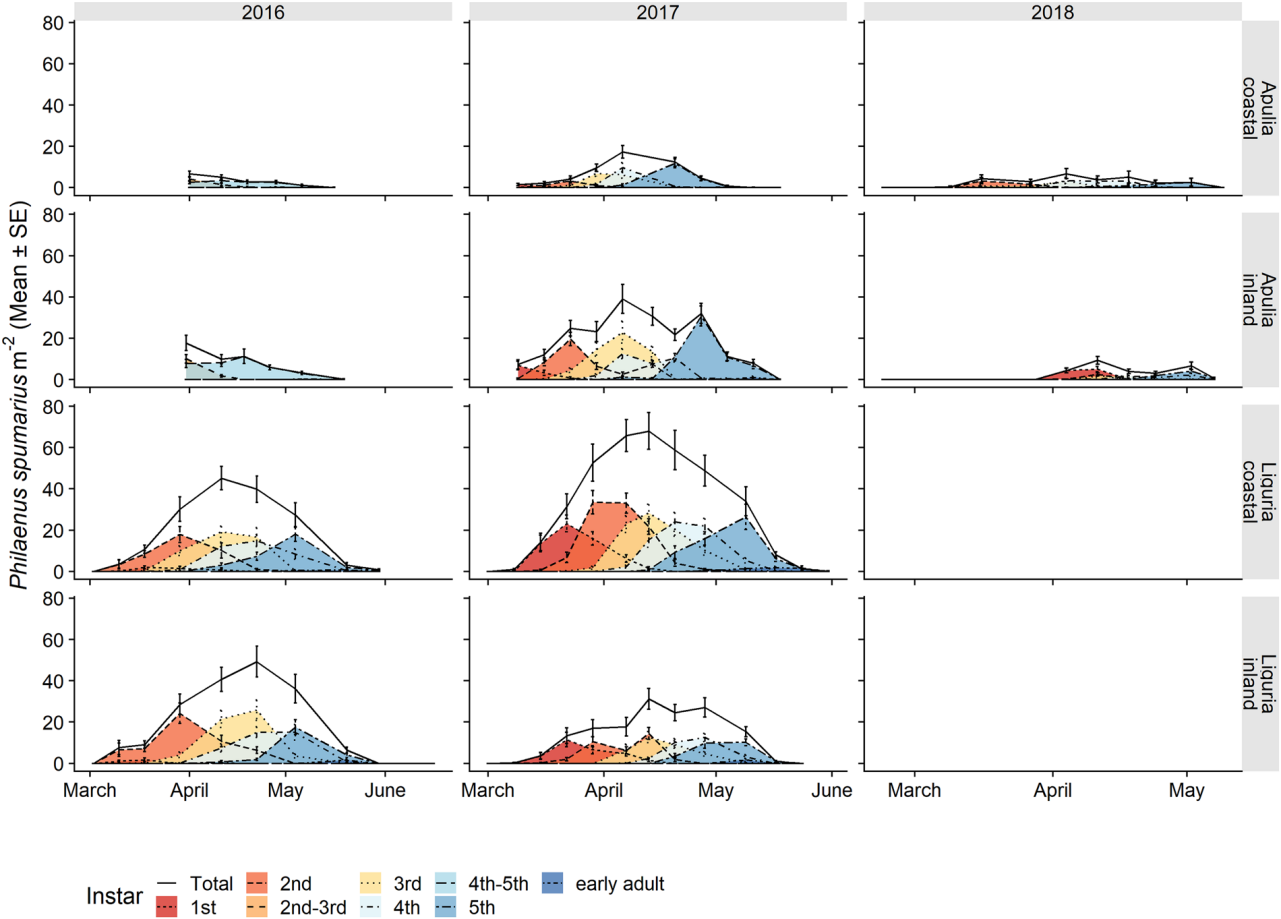
Life stage structure of the preimaginal populations of *Philaenus spumarius* in coastal and inland Mediterranean olive groves in Apulia and Liguria. The area curves of each nymphal instar, the total nymphs and the newly emerged adults are based on the mean densities, as estimated by quadrat sampling on each sampling date.

**Figure 3:**
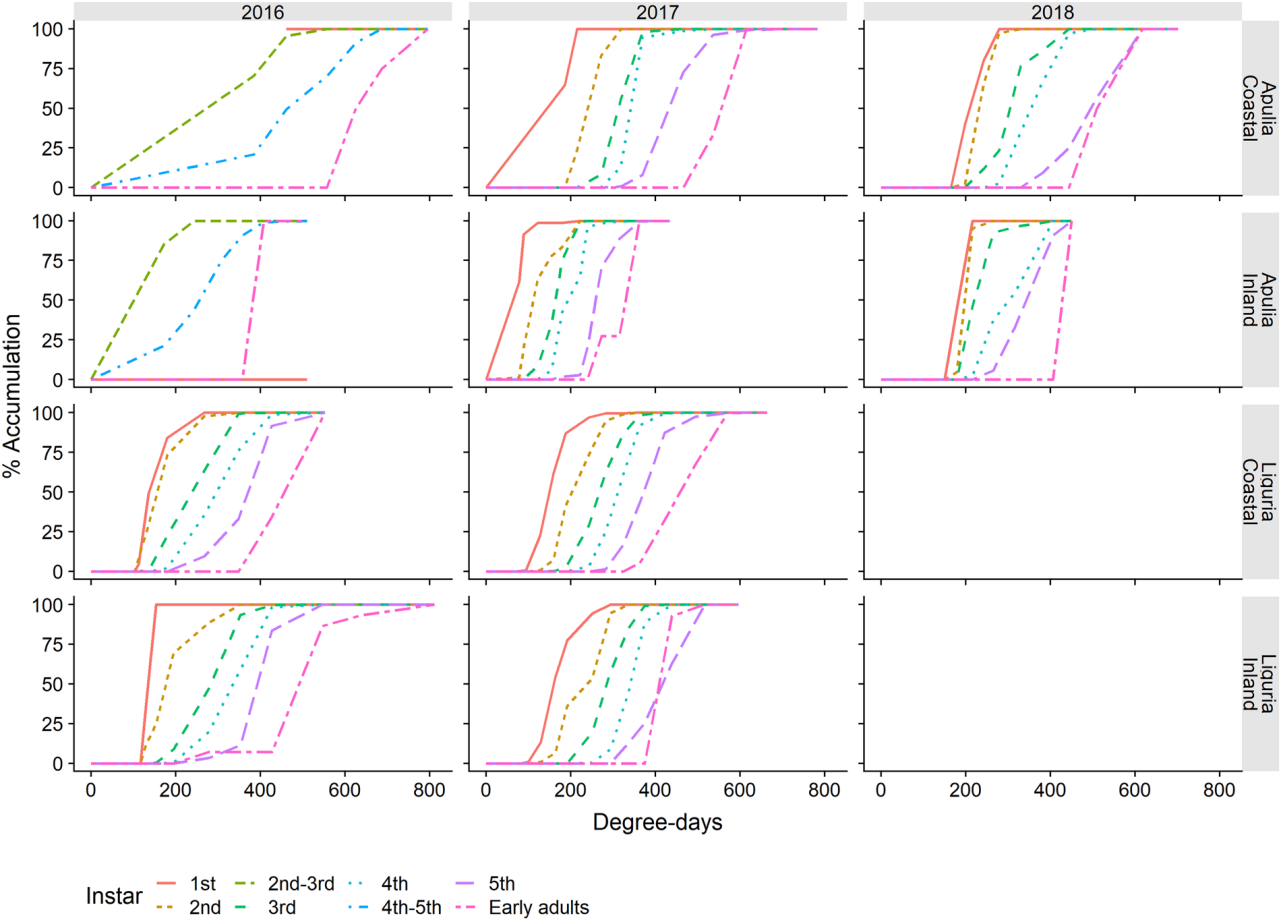
Observed cumulative population of the nymphal instars and newly emerged adults of *Philaenus spumarius* in relation to the accumulated degree-days in the coastal and inland Mediterranean olive groves in Apulia and Liguria.

*Philaenus spumarius* nymphs were detected, starting from the second week of March, with no significant chronological differences between the sampling years or olive groves monitored in Apulia and Liguria (Fig. 2 and Table 3). The physiological time of the first instar appearance varied from the lowest value of 78 DD in the Apulian inland site (Locorotondo) in 2017 to the highest value of 198 DD in the Apulian coastal site (Valenzano) in 2018 (Fig. 3). Less variability in the physiological time of the first instar appearance was recorded in Liguria, where it ranged from a minimum of 94 DD in the coastal site (Finale) in 2017 to a maximum of 100 DD in the inland site (Arnasco) in 2017. All the nymphal stages followed a similar pattern of appearance in the different olive groves to the one recorded for the 1^st^ instar for both the physiological (Fig. 3) and chronological timings (Fig. 2). The seasonal peak of nymphal populations was registered in mid-April in Liguria and slightly earlier in Apulia (Fig. 2 and Table 3). In Apulia, the lowest DD recorded for the nymphal population peak was 180 DD on 7^th^ April in Locorotondo in 2017 and the highest value was 369 DD on 7^th^ April in Valenzano in 2017. The data collected in Apulia during the 2016 surveys did not permit us to detect a clear population peak since the samplings started late, and the DD for the population peaks were not considered in 2016. In Liguria, the seasonal peak of the nymphal population ranged from a minimum of 267 DD on 11^th^ April in Finale in 2016 to a maximum of 294 DD on 13^th^ April in Arnasco in 2017. The nymphal densities declined gradually throughout late April and May, with the 5th nymphal stage being observed until the first half of May (2–19 May) in Apulia and until the second half of May (17–31 May) in Liguria (Table 3). In Apulia, the lowest DD for which the last nymphal instars were found was 434 DD, which was recorded on 19^th^ May in Locorotondo in 2017, whereas the highest DD in Valenzano was 689 DD and was recorded on 12^th^ May in 2017. In Liguria, the last nymphal instars were recorded at a minimum of 517 DD on 17^th^ May in Arnasco in 2017 to a maximum of 664 DD on 19^th^ May in Finale in 2017. Overall, in Liguria, the DD values for the first instar appearance, population peak and last nymphal instars, all fell within the Apulian DD range. The time span of the nymphal stages, i.e. from the appearance of the first nymphs to the appearance of the first adults, ranged from 30–50 days (195–350 DD) in Apulia to 50–70 days (269–417 DD) in Liguria (Table 3). The period of presence of each nymphal instar varied between 32 and 56–63 days in Liguria and between 14 and 36 days in Apulia, except for the first instar, which was collected over a limited time span of 2–3 weeks. The duration of each instar in Apulia was shorter for both the chronological and physiological times than in Liguria (Table 3 and Fig. 3). Overall, the early instar stages (1^st^-2^nd^-3^rd^) developed over higher cumulated DD in Liguria than in Apulia (Fig. 3).

**Table 3:** Dates of the first, last and peak occurrence of the different instars of *Philaenus spumarius* nymphs (peak dates = max no. of nymphs sampled).

		Liguria						Apulia						
		inland			coastal			inland			coastal			
		first	peak	last	first	peak	last	first	peak	last	first	peak	last	
2016	1st	10-Mar	18-Mar	18-Mar	10-Mar	18-Mar	11-Apr	—	—	—	11-Apr	11-Apr	11-Apr	
	2nd	10-Mar	29-Mar	22-Apr	10-Mar	29-Mar	22-Apr	31-Mar	31-Mar	11-Apr	1-Apr	1-Apr	19-Apr	
	3rd	18-Mar	22-Apr	20-May	18-Mar	11-Apr	4-May							
	4th	29-Mar	22-Apr	20-May	29-Mar	22-Apr	20-May	31-Mar	18-Apr	19-May	1-Apr	11-Apr		6-May
	5th	11-Apr	4-May	20-May	11-Apr	4-May	20-May							
	Ad	11-Apr	30-May	29-Nov	4-May	16-Jun	29-Nov	6-May	25-May	24-Oct	28-Apr	25-May	25-Oct	
2017	1st	8-Mar	22-Mar	13-Apr	8-Mar	22-Mar	28-Apr	10-Mar	10-Mar	15-Apr	10-Mar	10-Mar	17-Mar	
	2nd	15-Mar	13-Apr	20-Apr	15-Mar	29-Mar	28-Apr	10-Mar	24-Mar	15-Apr	17-Mar	24-Mar	31-Mar	
	3rd	7-Apr	13-Apr	9-May	22-Mar	13-Apr	17-May	17-Mar	7-Apr	21-Apr	24-Mar	31-Mar	21-Apr	
	4th	7-Apr	28-Apr	9-May	7-Apr	20-Apr	17-May	31-Mar	7-Apr	12-May	31-Mar	7-Apr	28-Apr	
	5th	20-Apr	9-May	17-May	13-Apr	9-May	31-May	7-Apr	28-Apr	19-May	31-Mar	21-Apr	12-May	
	Ad	9-May	5-Oct	6-Dec	28-Apr	24-May	19-Dec	28-Apr	19-May	14-Dec	28-Apr	19-May	19-Oct	
2018	1st	—	—	—	—	—	—	4-Apr	11-Apr	11-Apr	9-Mar	16-Mar	27-Mar	
	2nd	—	—	—	—	—	—	4-Apr	11-Apr	18-Apr	9-Mar	16-Mar	4-Apr	
	3rd	—	—	—	—	—	—	11-Apr	18-Apr	2-May	16-Mar	4-Apr	18-Apr	
	4th	—	—	—	—	—	—	11-Apr	2-May	2-May	27-Mar	4-Apr	24-Apr	
	5th	—	—	—	—	—	—	18-Apr	2-May	7-May	11-Apr	2-May	2-May	
	Ad	—	—	—	—	—	—	2-May	31-May	16-Oct	24-Apr	12-Jun	30-Oct

The mean densities of the nymphal stages were higher in Liguria, with a range of population peaks of 31–68 nymphs m^−2^, compared to the Apulia peaks, which ranged from 7 to 39 nymphs m^−2^ (Fig. 2). The progression of juvenile stages of *P. spumarius* occurred as a series of highly overlapping distributions that extended approximately over three months during spring, both in Apulia and Liguria. Indeed, several different nymphal instars were found on each sampling date, and the 1^st^ instar only disappeared when the 4^th^ instar was present. The maximum nymphal population densities were relatively constant for the different instars within each olive grove, but they varied between the olive groves, regions and years. For example, the overall densities of *P. spumarius* registered in Apulia during the 2018 surveys were much lower than those found in 2017 in the same sites (the maximum density in Locorotondo was 39.1 nymphs m^−2^ in 2017 and 9.3 nymphs m^−2^ in 2018) (Fig. 2). The first instar was an exception, because its densities were always low, probably because of the difficulty detecting these small nymphs under field conditions and its shorter development time than that of the other nymphal stages.

The temporal occurrence of *P*. spumarius nymphs followed a well-defined pattern in all of the monitored olive groves, that is, emerging in early March, peaking in April, and emerging to the adult stage from late April until the end of May. The temperatures registered in late winter and spring were quite similar in the investigated olive groves in both regions, although lower temperatures were recorded in the inland olive grove in Apulia than in the coastal one (see Supplementary Fig. S2). The slight difference in the phenology of spittlebugs in the two Mediterranean regions was therefore not completely unexpected, since the hatching and development of *P. spumarius* nymphs are influenced by the temperature and photoperiod, although some contrasting results have been reported^23,41,52,53^. The differences in DD needed the for nymphal development of *P. spumarius* observed in the surveyed olive groves were related to: i) the non-linear responses of the development rate at high temperatures and a rather low value for the upper development threshold, (see Chmiel and Wilson^32^, who estimated a nymphal upper threshold of 26.7 °C), ii) the gradual egg hatching, possibly influenced by differences in cold temperatures experienced throughout winter^23^. Further experiments focused on disentangling the effect of the temperature on juvenile development are currently ongoing in our laboratory under controlled conditions to control for any confounding effects, such as host plant communities or population differences.

Our results on the *P. spumarius* nymphal stage phenology are consistent with some that have recently been reported for other Mediterranean olive groves^9,43,44^. In previous studies carried out in the USA, a similar development timing was reported for California, whose climatic conditions are similar to the Mediterranean ones, whereas a delay of almost one month was registered in other States^29,32,54,55^. Some studies conducted in Europe, in the UK^22,56^, Germany^23^ and the Black Sea region of Turkey^57^, investigated the phenology of the meadow spittlebug in northern regions and found nymphal stages between April and June. According to Halkka *et al*.^21^, the chronological delay in nymph development in Finland, compared to the Mediterranean basin, exceeds two months.

#### Adult stage

Newly emerged adults were detected from late April to early May. Adult emergence occurred without any relevant differences in chronological time between the olive groves and regions. In 2016, in the inland grove of Liguria, a single freshly emerged adult was already found on 11^th^ April (278 DD), 3 weeks earlier than the first ones observed in the coastal grove on 4^th^ May (426 DD). However, adults only started to emerge consistently on 20^th^ May, 71 days after the first observation of nymphs at 547 DD. In 2017, in Locorotondo, the first adults emerged at 273 DD on 28^th^ April, which is the lowest DD recorded at either site, while the highest DD recorded for first adult emergence was 626 DD on 28^th^ April in Valenzano in 2016 (Fig. 3).

**Figure 4:**
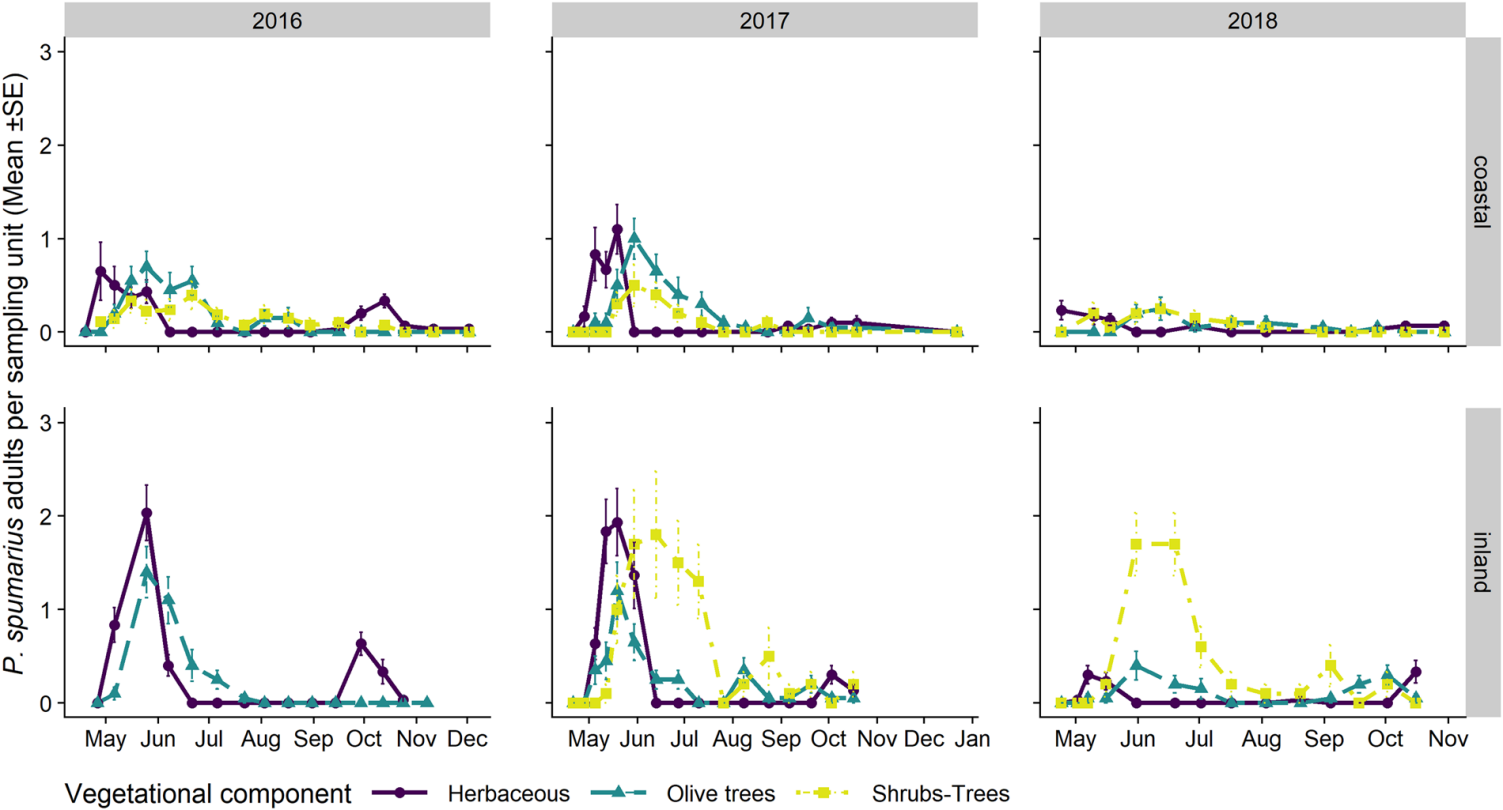
Mean (±SE) number of *Philaenus spumarius* adults per sampling unit during the 2016–2018 period in the Apulia olive groves on three different vegetational compartments: herbaceous cover (solid line/point), olive trees (dashed line/triangle) and wild woody plants (dashed dotted line/square). Note that the sampling units differ among vegetational types, that is, 4 sweeps on the herbaceous cover and ten on the olive trees and wild woody plants.

**Figure 5:**
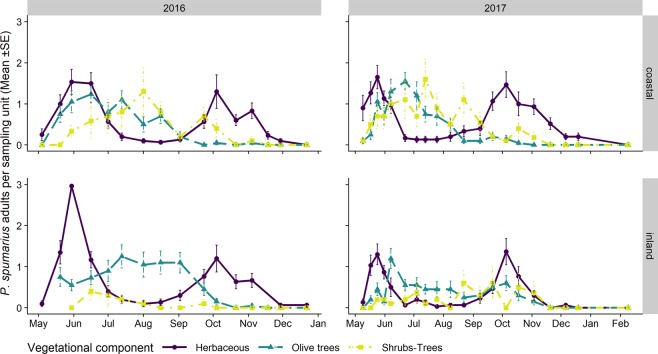
Mean (±SE) number of *Philaenus spumarius* adults per sampling unit collected by means of sweeping nets during the 2016–2017 period in the Liguria olive groves on three different vegetational compartments: herbaceous cover (solid line/point), olive trees (dashed line/triangle) and wild woody plants (dashed dotted line/square). Note that the sampling units differ among vegetational types, that is, 4 sweeps on the herbaceous cover and ten on olive trees and wild woody plants.

Following emergence, *Philaenus spumarius* adults were found in high number on herbaceous cover in late May-mid June, in both the Apulian and Ligurian olive groves (Figs. 4, 5). The peak of adult abundance in spring in Apulia ranged from 434 DD in Locorotondo in 2017 to 1213 DD in Valenzano in 2018 (Table 4). In Liguria, the peak of adult population abundance in spring ranged from 571 DD in Finale in 2017 to 822 DD in Finale in 2016. Up to 1.55 ± 0.22 adults per olive tree were collected on olive canopies during late spring, about 1–3 weeks later than the abundance peak found on the herbaceous cover (Figs. 4 and 5).

**Table 4:** First, last and peak dates and degree-days (DD) of the *Philaenus spumarius* adult stage (peak dates = max no. of adults sampled).

		Liguria						Apulia					
		inland			coastal			inland			coastal		
		first	peak	last	first	peak	last	first	peak	last	first	peak	last
2016	day	11-Apr	30-May	29-Nov	4-May	16-Jun	29-Nov	6-May	25-May	24-Oct	28-Apr	25-May	25-Oct
	DD	278	638	2791	426	822	2846	408	560	2491	626	888	3203
2017	day	9-May	5-Oct	6-Dec	28-Apr	24-May	19-Dec	28-Apr	19-May	14-Dec	28-Apr	19-May	19-Oct
	DD	439	2604	2967	363	571	2942	273	434	—	536	784	3350
2018	day	—	—	—	—	—	—	2-May	31-May	16-Oct	24-Apr	12-Jun	30-Oct
	DD	—	—	—	—	—	—	406	697	2673	510	1213	3421

The density of *P. spumarius* adults decreased in the entire olive agroecosystem during summer, although some differences were observed between Apulia and Liguria. In the Apulian olive groves, the number of *P. spumarius* decreased abruptly on the herbaceous cover from late spring, with no spittlebugs being sampled from mid-June until mid-September (Fig. 4), in part as a consequence of soil tilling. The spittlebug populations on woody plants (both olives and wild trees) decreased more slowly than on the herbaceous cover, and a limited presence of individuals was registered throughout the summer. In Liguria, *P. spumarius* did not disappear from the herbaceous cover during summer, and adults were present with relatively high densities on olive canopies (mean range 0.5–1 adult/tree) and, especially in coastal olive grove, on wild woody hosts (up to 1.6 adult/tree) (Fig. 5). The abundance and the pattern of *P. spumarius* registered on wild woody hosts differed greatly between the olive groves. These differences could probably be explained by considering the diverse availability of alternative host plants for the spittlebugs and the heterogeneous spatial structure of the considered olive groves. For instance, the densities of *P. spumarius* in the coastal olive grove of Liguria were high on shrubs and trees, and were similar to those registered on the herbaceous cover, while only a few meadow spittlebugs were collected on wild woody hosts throughout the entire sampling season in the inland olive grove. As far as the preference of *P. spumarius* for woody plants is concerned, the highest abundance was reported on *Quercus* spp., *Pistacia lentiscus* L. and *P. terebinthus* L. in Liguria and on *Quercus* spp. and *Myrtus communis* L. in Apulia.

In late summer, the number of adults on the herbaceous cover tended to increase again, probably due to the return of spittlebugs from aestivation sites (e.g. wild woody hosts inside and outside the agroecosystems). This return was considerable, particularly in the Liguria olive groves, where similar spittlebug densities to those observed in spring were recorded in autumn (mean density of up to 1.47 adults/SSU_h_). The densities of *P. spumarius* in Apulia were lower (mean value up to 0.33 adults/SSU_h_), probably because of a higher adult mortality rate during summer (Figs. 4 and 5). The overall number of adults sampled in the olive grove agroecosystem decreased again in late October or early November, with very few individuals being recorded in late autumn. The last adults in Liguria were sampled in late November or December. In Apulia, the samplings had often ended by late October, even though a few spittlebugs were still present in the olive groves. Indeed, some *P. spumarius* adults were collected in the same olive groves in late winter, thus proving that a few adults may overwinter in South Italy (Dr. V. Cavalieri, unpublished data).

The population peaks of the adult stage were quite similar in the Apulia and Liguria regions, although there were important differences between the olive groves. The differences in *P. spumarius* density between the two regions might be due to several reasons, amongst which: i) intrinsic differences in suitability for spittlebugs of specific olive groves, considering the surrounding habitats, i.e. landscape composition, which can provide alternative habitats for spittlebug adults, thus contributing towards maintaining high population levels of vectors in olive groves through seasonal mass movements; ii) different levels of insect disturbance, as a result of agronomic measures, such as soil tillage –which is usually carried out in summer in Apulia – can have an impact on the adult population, thereby determining mass movements to olive trees and other woody hosts, and may influence the oviposition suitability of certain sites, thus impacting the following year’s juvenile populations. Given the different ecological situation of the sites inspected in the Liguria and Apulia regions, we hypothesize that the *P. spumarius* population abundance was higher in Liguria mainly because of the landscape structure (much more fragmented in Liguria) and the richness of non-cultivated refuge areas, which can represent a more suitable environment for spittlebugs^58^. Again in this case, the more intensive agricultural exploitation of the Bari province area, both at a landscape level and because of the use of more intense agronomic measures, could account for the lower population level of the spittlebug registered in our study compared to that found in previous studies performed in the Lecce Province (South Apulia)^44,59^.

#### Sex ratio

**Figure 6:**
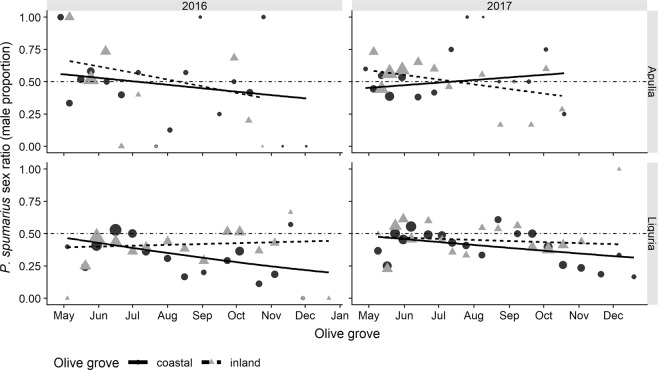
Sex ratio of *Philaenus spumarius* adults as the proportion of males sampled in Apulia and Liguria olive groves during 2016, 2017, (2018). Black dots (●) represent coastal olive groves and grey triangles  inland olive groves. The size of the symbols is proportional to the number of insects sampled on that date; the solid line represents fitted values for coastal olive groves and the dashed line indicates fitted values for inland olive groves (GLM binomial model, see Table 5). The horizontal dash-dotted line shows sex ratio equality (0.5).

The sex ratio of *P. spumarius* adults, determined over the whole season, was slightly male biased in the Apulian olive groves (male proportion = 0.54), while it was female biased in the Ligurian sites (male proportion = 0.41). A significantly greater proportion of males was recorded during the adult emergence period, especially in Apulia. The proportion of males tended to decrease during the adult season in all the olive groves, until a parity of male / female proportions was reached at the end of the emergence period (mid-May) (Fig. 6). Subsequently, the proportion of males continued to decrease throughout the year, although it fluctuated to a great extent during summer, with a few or no *P. spumarius* males being collected on the last sampling dates. No differences were registered among the vegetation compartments. Therefore, the sex ratio varied slightly, although it was statistically significant, between the regions, olive groves and sampling dates, but with no significant interactions (Table 5). Males appeared earlier in spring, probably because their preimaginal development was faster than that of the females, as already reported in previous studies^21,60^. The sex ratio became female biased throughout the summer season, probably because of a shorter longevity of the males^29,61,62^. Overall, the male proportion was significantly higher in Apulia than in Liguria. A different prevalence of the sex-manipulating proteobacteria *Wolbachia* in the spittlebug populations of the two Regions might account for the local sex ratio differences^63^.

**Table 5:** Effect of the Region, olive grove position (coastal/inland) and sampling date (days after first adult sampling) on the sex ratio (proportion of males) of *Philaenus spumarius* adults.

Explanatory variables	Sum Sq	*Df*	χ^2^	P value
Sex ratio				
Region	323.9	1	16.56	**<0.001**
Year	7.1	2	0.34	0.842
Vegetation compartment	5.4	2	0.27	0.872
Olive grove position	105.8	1	5.41	**0.02**
Time after first sampling (days)	157.9	1	8.07	**0.004**
Residuals	5729.8	293		

Interactions are not reported since they were not significant and were discarded during the model selection. GLM with a quasi-binomial error structure, data on sex ratio were pooled according to the sampling date.

### Other spittlebug species

**Figure 7:**
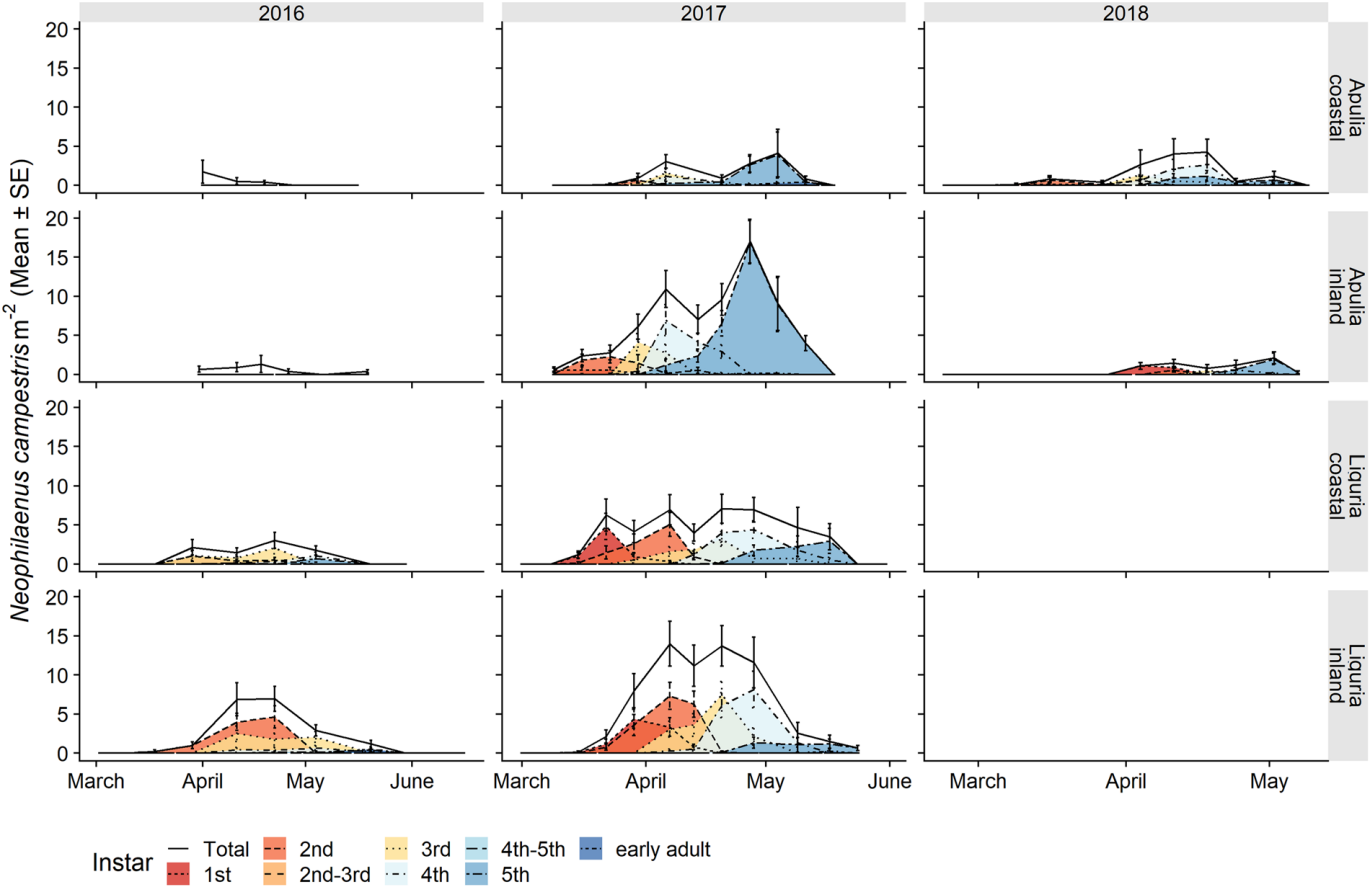
Life stage structure of the preimaginal populations of *Neophilaenus campestris* in coastal and inland Mediterranean olive groves in Apulia and Liguria. The area curves for each nymphal instar, the total nymphs and newly emerged adults are based on the mean densities, estimated by means of quadrat sampling on each sampling date.

**Figure 8:**
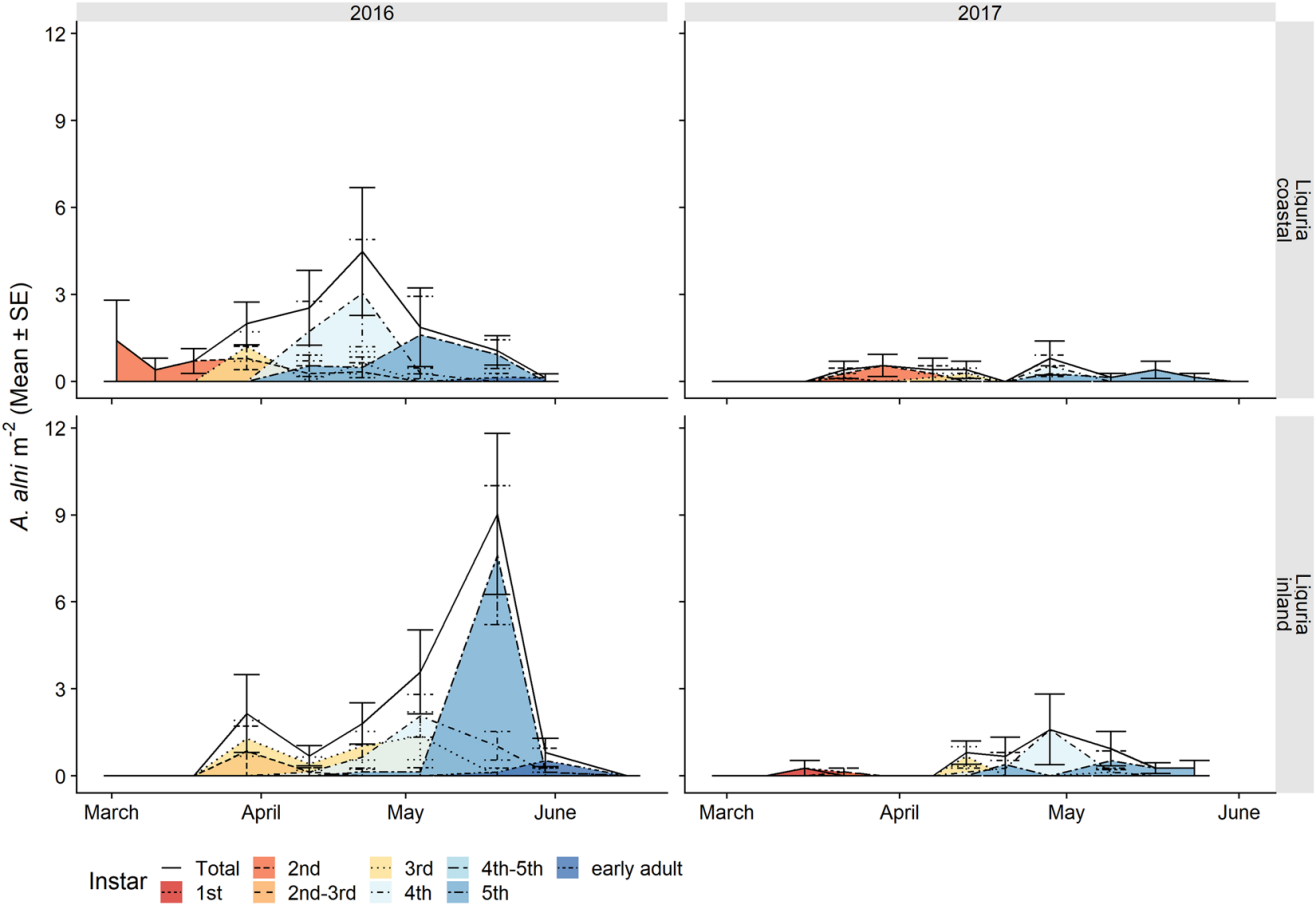
Life stage structure of the preimaginal populations of *Aphrophora alni* in coastal and inland Mediterranean olive groves in the Liguria region. The area curves for each nymphal instar, the total nymphs and newly emerged adults are based on the mean densities, estimated by means of quadrat sampling on each sampling date.

The nymphal populations of *N. campestris* followed a similar trend as *P. spumarius*, although lower abundance levels were observed. The mean peak densities ranged between 1 and 16 nymphs m^−2^. The highest nymphal density was registered in 2017 in the inland olive groves in both Apulia and Liguria. The nymphal stages spanned from mid-March to May, and peaked during April (Fig. 7 and Table 6). Adults started to emerge earlier in Apulia, that is, mid to Late-April, compared to Liguria, where they started to emerge mid-May (Table 6). *Aphrophora alni* juveniles were collected from mid-March to late May on the herbaceous ground cover, albeit only in the Ligurian olive groves (Table 7). The population densities were lower than 2 individuals m^−2^ throughout spring, except for a 5^th^ instar peak in late May 2016 in the inland olive grove (Arnasco), which reached 7.61 ± 2.40 nymphs m^−2^. The mean density of the *A. alni* nymphs in the two Ligurian olive groves was higher in 2016 (1.05 ± 0.16 nymphs m^−2^) than in the 2017 survey (0.17 ± 0.04 nymphs m^−2^) (Fig. 8).

**Table 6:** Dates of the first, last and peak occurrences of the different instars of *Neophilaenus campestris* nymphs (peak dates = max no. of *nymphs* sampled).

Year	Instar	Liguria							Apulia					
		inland				coastal			inland			coastal		
		first	peak	last		first	peak	last	first	peak	last	first	peak	last
2016	1st	—	—	—		—	—	—	—	—	—	—	—	—
	2nd	18-Mar	22-Apr	22-Apr		29-Mar	29-Mar	22-Apr	31-Mar	11-Apr	11-Apr	1-Apr	1-Apr	1-Apr
	3rd	11-Apr	11-Apr	4-May		29-Mar	22-Apr	22-Apr						
	4th	11-Apr	4-May	20-May		11-Apr	4-May	4-May	11-Apr	18-Apr	19-May	11-Apr	11-Apr	19-Apr
	5th	22-Apr	20-May	20-May		11-Apr	4-May	4-May						
	Ad	20-May	21-Oct	29-Nov		20-May	4-Nov	29-Nov	25-May	25-May	12-Oct	16-May	25-May	29-Sep
2017	1st	15-Mar	29-Mar	13-Apr		15-Mar	22-Mar	7-Apr	10-Mar	17-Mar	31-Mar	24-Mar	24-Mar	24-Mar
	2nd	22-Mar	7-Apr	20-Apr		15-Mar	7-Apr	13-Apr	10-Mar	24-Mar	15-Apr	24-Mar	31-Mar	31-Mar
	3rd	7-Apr	20-Apr	28-Apr		29-Mar	20-Apr	9-May	31-Mar	31-Mar	21-Apr	31-Mar	7-Apr	21-Apr
	4th	7-Apr	28-Apr	9-May		13-Apr	28-Apr	17-May	31-Mar	7-Apr	21-Apr	7-Apr	7-Apr	21-Apr
	5th	20-Apr	28-Apr	24-May		20-Apr	17-May	17-May	7-Apr	28-Apr	12-May	7-Apr	5-May	12-May
	Ad	17-May	21-Sep	5-Oct		9-May	21-Sep	5-Oct	28-Apr	19-May	13-Jun	28-Apr	19-May	19-Oct
2018	1st	—	—	—	—	—	—	—	4-Apr	4-Apr	11-Apr	9-Mar	16-Mar	27-Mar
	2nd	—	—	—	—	—	—	—	11-Apr	11-Apr	11-Apr	16-Mar	16-Mar	4-Apr
	3rd	—	—	—	—	—	—	—	18-Apr	18-Apr	18-Apr	27-Mar	4-Apr	18-Apr
	4th	—	—	—	—	—	—	—	18-Apr	24-Apr	2-May	4-Apr	18-Apr	2-May
	5th	—	—	—	—	—	—	—	24-Apr	2-May	7-May	11-Apr	18-Apr	2-May
	Ad	—	—	—	—	—	—	—	7-May	31-May	16-Oct	24-Apr	10-May	30-Oct

**Table 7:** Dates of the first, last and peak occurrences of the different instars of *Aphrophora alni* (peak dates = max no. of insects sampled).

Year	Instar	Liguria					
		inland			coastal		
		first	peak	last	first	peak	last
2016	1st	—	—	—	—	—	—
	2nd	11-Apr	29-Mar	4-May	18-Mar	2-Mar	22-Apr
	3rd	29-Mar	4-May	30-May	29-Mar	29-Mar	22-Apr
	4th	11-Apr	4-May	30-May	11-Apr	22-Apr	4-May
	5th	22-Apr	20-May	30-May	22-Apr	4-May	20-May
	Ad	20-May	1-Jul	4-Oct	20-May	30-May	21-Oct
2017	1st	22-Mar	15-Mar	22-Mar	22-Mar	22-Mar	22-Mar
	2nd	22-Mar	22-Mar	22-Mar	22-Mar	29-Mar	7-Apr
	3rd	13-Apr	13-Apr	9-May	7-Apr	13-Apr	13-Apr
	4th	13-Apr	28-Apr	9-May	13-Apr	28-Apr	28-Apr
	5th	9-May	20-Apr	24-May	9-May	17-May	24-May
	Ad	24-May	7-Jun	18-Oct	17-May	24-May	21-Sep

**Figure 9:**
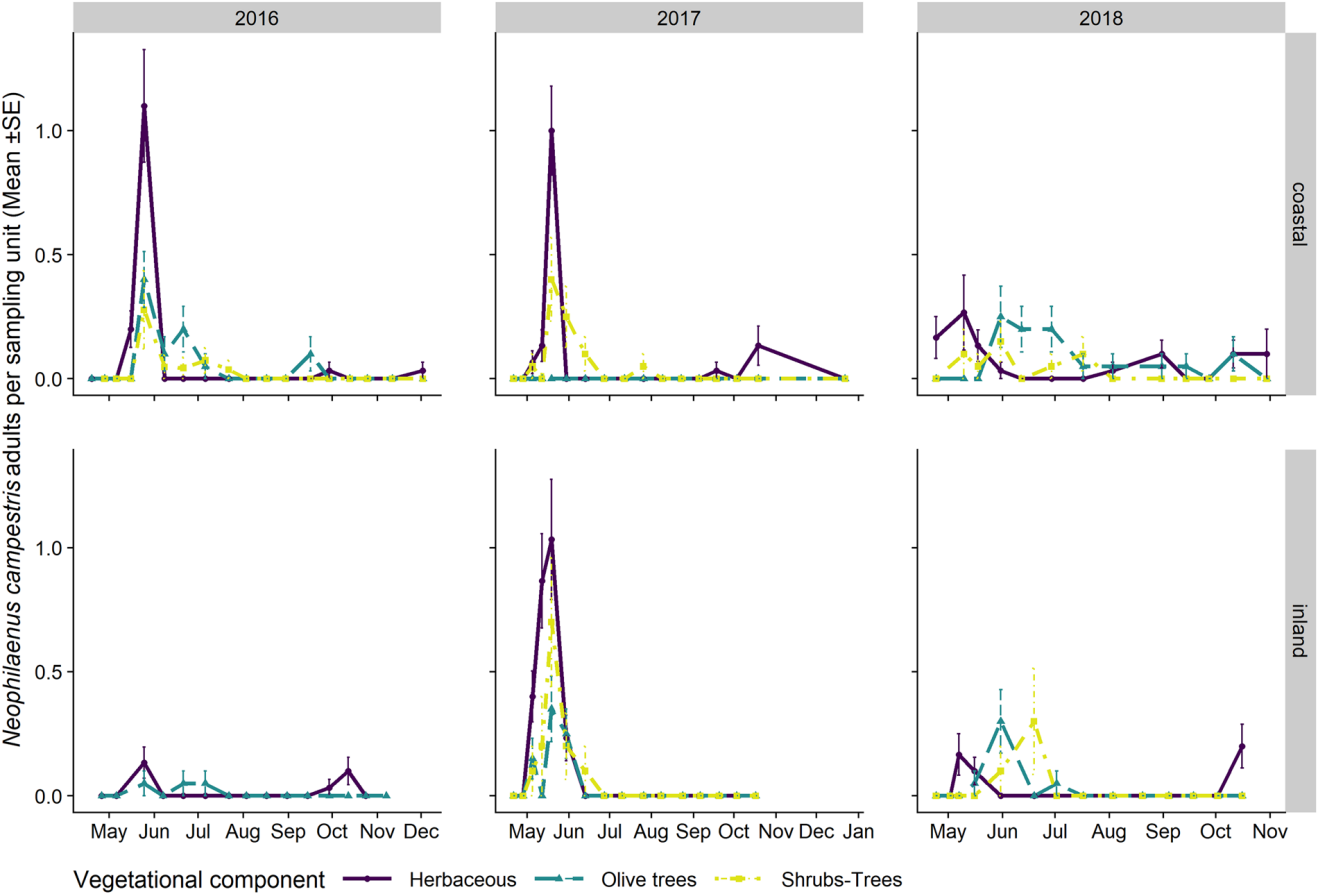
Mean (±SE) number of *Neophilaenus campestris* adults per sampling unit sampled by means of sweeping nets during the 2016–2018 period in the Apulia olive groves on three different vegetational compartments: herbaceous cover (black solid line/point), olive trees (grey dashed line/triangle) and wild woody plants (grey dashed dotted line/square). Note that the sampling units differ among vegetational types, that is, 4 sweeps on the herbaceous cover and ten on olive trees and wild woody plants.

**Figure 10:**
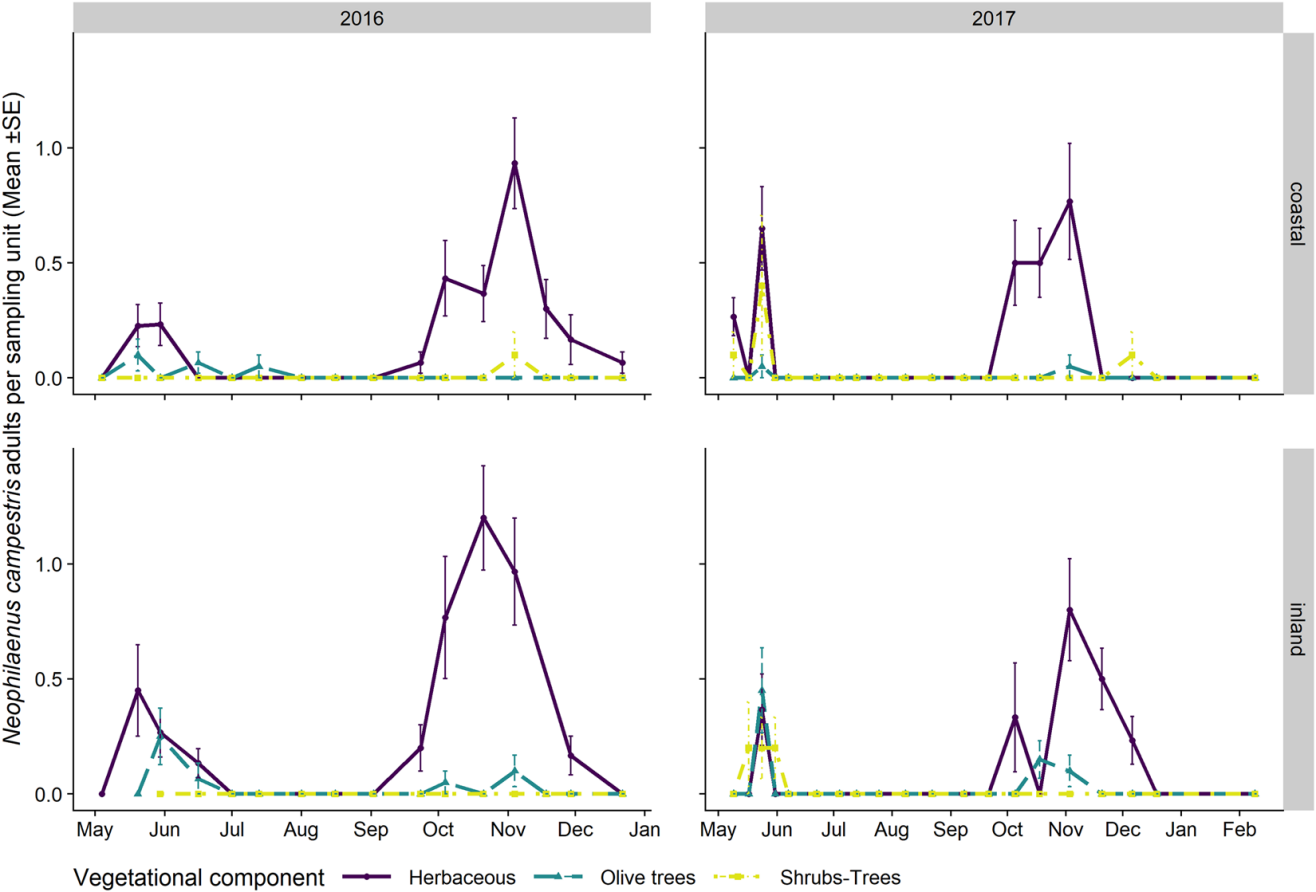
Mean (±SE) number of *Neophilaenus campestris* adults per sampling unit sampled by means of sweeping nets during the 2016–2018 period in Liguria olive groves on three different vegetational compartments: herbaceous cover (black solid line/point), olive trees (grey dashed line/triangle) and wild woody plants (grey dashed dotted line/square). Note that the sampling units differ among vegetational types, that is, 4 sweeps on the herbaceous cover and ten on olive trees and wild woody plants.

**Figure 11:**
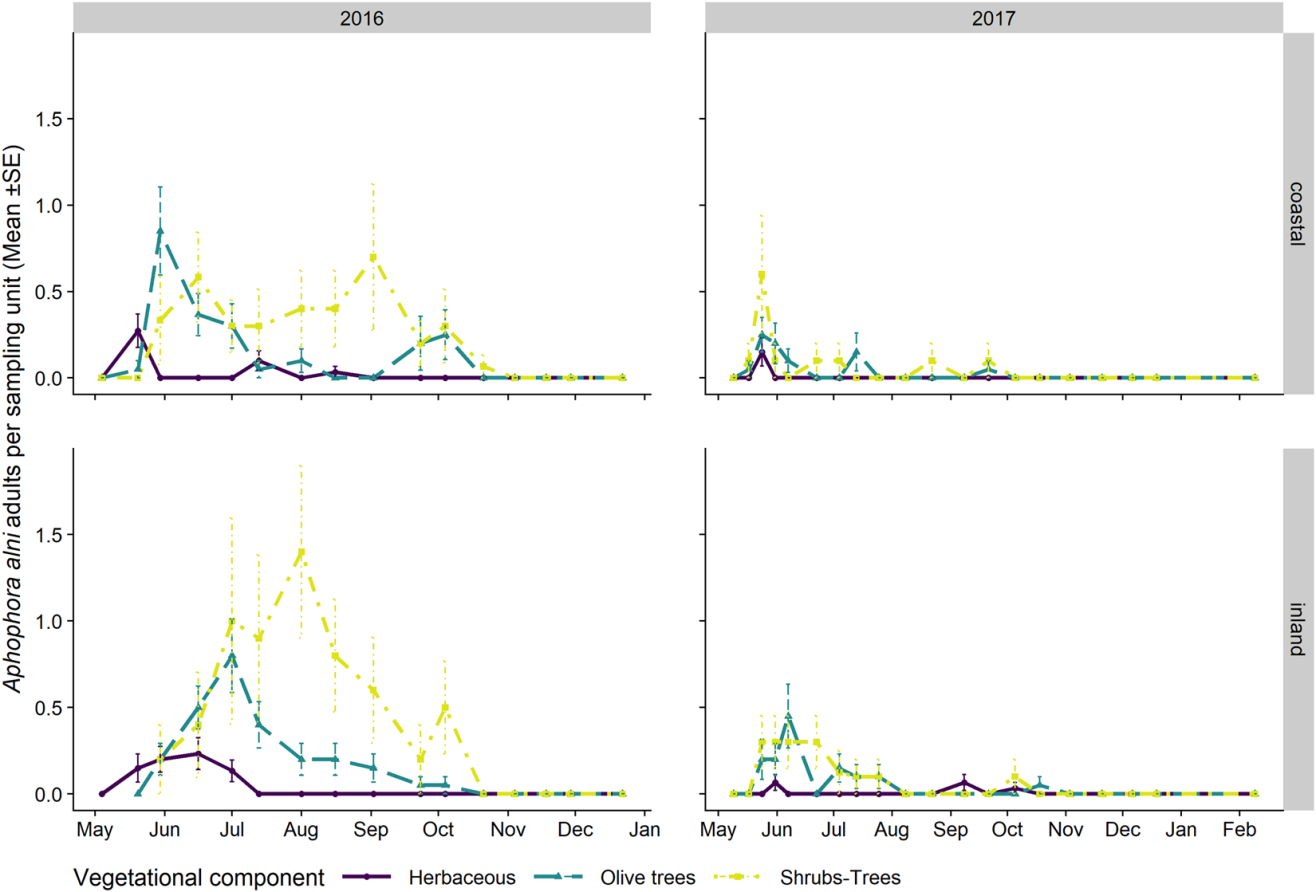
Mean (±SE) number of *Aphrophora alni* adults per sampling unit sampled by means of sweeping nets during the 2016–2018 period in Liguria olive groves on three different vegetational compartments: herbaceous cover (black solid line/point), olive trees (grey dashed line/triangle) and wild woody plants (grey dashed dotted line/square). Note that the sampling units differ among vegetational types, that is, 4 sweeps on herbaceous cover and ten on olive trees and wild woody plants.

The adult populations of *N. campestris* followed a different pattern throughout the year from *P. spumarius*. Adults were mainly collected in the herbaceous cover, especially where grasses (i.e. Poaceae) were dominant. The highest abundances of *N. campestris* adults were registered on the herbaceous cover in Apulia in late May, soon after the end of the nymphal stages, whereas the highest population levels in Liguria were registered during autumn. *N. campestris* adults were not usually found during summer in any of the vegetation compartments of the olive agroecosystem in either Apulia or Liguria (Figs. 9 and 10). Such an absence of *N. campestris* in the olive groves is probably due to the movement of spittlebugs to aestivation host plants, that is, usually conifer trees^64^. *Aphrophora alni* adults were only sampled in Liguria, mainly on woody plants, on both olive trees and wild broadleaved trees (e.g. *Quercus* spp.). The population abundance levels were higher in 2016 than in 2017 in both olive groves (the mean number of individuals collected per sampling date in 2016 was 14.4 ± 3.70, while it was 3.85 ± 1.30 in 2017). The highest densities on olive trees were registered in June (up to 0.85 ± 0.25 adults/olive) and they then decreased during summer. The populations on wild woody plants peaked later, in July/August, with up to 1.4 ± 0.50 adults/tree. In 2016, the *A. alni* density on wild trees remained quite constant until late summer. A few individuals were occasionally sampled in autumn (Fig. 11).

*Neophilaenus campestris* showed lower population levels than *P. spumarius* in both regions for both nymphs and adults, as already reported for other olive groves in Apulia^44,59^ and in Greece^41,42^. Conversely, *N. campestris* has been found to be the predominant spittlebug species in olive groves in Spain, although dramatic fluctuations of population levels have been observed between olive orchards^43^. The chronological time of nymphal instars or adult appearance of *N. campestris* was quite similar to those of *P. spumarius*, although a certain time lag of the appearance of *N. campestris* nymphs was registered (Fig. 7). Such a delay could have partly been caused by the lower abundance of the former, and thus by the lower probability of sampling its nymphs at the very beginning of nymphal development^65^. Moreover, the onset of nymphal development of *A. alni* was unclear, given the low densities that were observed, but adults only started to emerge in late May, later than *P. spumarius*. To the best of our knowledge, no other information is available on the nymphal instar phenology of *N. campestris* and *A. alni*. Only a few investigations have been carried out on some *Neophilaenus* species in the UK^34,53,65^ and in Germany^38^, in which nymphal development between late April and mid-June has been reported, that is, about one month later than for *N. campestris* under Mediterranean conditions.

### Conclusions

A three-year study has been conducted under field conditions in order to provide a detailed description of the population dynamics, abundance and phenology of spittlebugs in Mediterranean olive groves. The study contributes to fill the knowledge gap on the ecology and biology of this group of insects and to pave the way towards a better understanding of the epidemiology of *Xf*-associated diseases in the Mediterranean basin, particularly in olive groves. Different xylem feeder species, all belonging to the Cercopoidea superfamily (spittlebugs) were collected during the three-year field survey. No sharpshooters (Hemiptera: Cicadellidae Cicadellinae) were found in our sampling campaigns. This result confirms what has already been reported for other olive groves in the Mediterranean region^41,43,59^. However, the presence of sharpshooter species in some olive agroecosystems cannot be ruled out. Indeed, a few *Cicadella viridis* L. adults have been collected in olive groves in the Italian Abruzzo^66^ and Veneto Regions (Nicola Mori, Personal communication), and in Turkey^67^. In our study, two xylem-sap feeder species were predominant, *P. spumarius* and *N. campestris*. A third spittlebug species, *A. alni*, was only present in the Liguria olive groves. Three other spittlebug species, *L. coleoptrata*, *C. vulnerata* and *C. sanguinolenta*, were also recorded, albeit all in low numbers and for a restricted time span. Although olive groves represent a rather biodiverse agroecosystem, as the olive is a perennial crop and the grove has a ground cover of herbaceous plants, the richness of spittlebug species in olive groves seems to be limited. Data from other surveys carried out in olive groves^40–42,59,68^ and in vineyards^69,70^ also indicate the presence of a limited biodiversity of this taxon under cropping systems, compared to biodiversity registered in more natural grassland habitats in Northern Europe^37,71,72^.

Our results on the phenology and abundance of the nymphal and adult stages of spittlebugs provide useful indications for vector control. The description of stage-structured populations of nymphs and adults allows to identify the best time to apply control. According to our observations, the newly hatched nymphs (1^st^ instar) always disappeared just before the peak of the 4^th^ instar nymphs, and the first adults were only sampled after this peak. Therefore, any control measure applied at the 4^th^ instar peak could potentially target the whole nymphal population before the onset of the adults, thus achieving the maximum efficacy. A first abundance peak was observed for the adults soon after emergence in all the olive groves on both the herbaceous cover and on the olive trees. These considerations, which are similar to those of Cornara *et al*.^9^, suggest that the period immediately after adult emergence is the crucial moment for both *X. fastidiosa* acquisition and transmission to olives by insect vectors. It is worth pointing out that, once infected, vectors are persistently infectious. Insecticides targeting the adult stage should be applied timely to the olive canopy, mainly in this period, in order to prevent the spread of the disease during the year^11,73,74^. Hence, transmission may occur all over the summer and at the beginning of autumn, even though a limited presence of adults was recorded on olive canopies later in the season. In Liguria − a *Xf*-free area − *P. spumarius* adults were collected in relatively high numbers on olives from their emergence to the end of September, as has also been noted in other regions in Northern Italy (Pavan^27^ and authors’ unpublished data). Indeed, where the summer conditions are less severe (e.g. Northern Europe), spittlebug adults can be collected over the whole summer under cropping and non-cropping systems^21,65,75^. The extended period of the presence of *P. spumarius* on olive trees in Liguria, together with the high populations of recorded spittlebugs, suggests that the eventual introduction of a strain of *X. fastidiosa* infecting olive plants in this area could potentially lead to a dramatic outbreak, although vectors are only one of the actors involved in epidemics. Furthermore, the difference in composition of spittlebug communities in Mediterranean olive groves could influence the epidemiology of *X. fastidiosa*. Mediterranean regions with lower population levels of *P. spumarius* than those of *Neophilaenus* spp, e.g. Spain^43,64^, could present less inoculum pressure on olive trees and a subsequent epidemic spread of the bacterium, since *Neophilaenus* spp. are rarely collected on olive canopies, as demonstrated in this study and others^10,42^.

The summer colonisation of wild shrubs/trees inside and around the olive agroecosystem by spittlebugs, and by *P. spumarius* and *A. alni* in particular, highlights the role of these plants as reservoirs of potential vector insects. Some of these plants, e.g. *Quercus ilex, Rhamnus alaternus*, *Laurus nobilis, Phyllirea latifolia* and *Myrtus communis*, can also act as *X. fastidiosa* ST53 reservoirs^10^. The colonisation of these alternative host plants by spittlebugs is possible as they are highly polyphagous, and wild plants might be more suitable (e.g. less water stressed) during the dry summer months than olive trees. The aestivation period and/or local migration towards natural or semi-natural patches of these wild host plants could occur, as already noticed in other hot Mediterranean areas^18,41,43^. In autumn, the adult populations increased on the herbaceous ground cover, which represents the oviposition site, thus confirming that most of the spittlebugs did not die during summer. The spatial distribution of spittlebugs throughout the season, inside and outside the olive groves, needs dedicated studies, since knowledge on this topic could contribute towards the understanding of the spread of epidemics and help in the designing of an effective management of vector populations^76^. Similarly, the investigation of the role of the landscape composition may help predict *P. spumarius* population levels in specific areas^58^. Such a prediction is needed to assess the risk to new plantings of *Xf*-susceptible crops in infected areas, as well as to evaluate the probability of the establishment and spread of the bacterium in the case of its introduction into new areas.

The findings of our study underline the great variability of population levels of potential *X. fastidiosa* vectors in olive groves between and within different Mediterranean regions. There is still a general lack of information on both the abiotic and biotic factors that influence the composition of xylem-sap feeder communities in olive groves and on species abundance. Further studies on vectors in olive, almond and other agroecosystems potentially at risk to the spread of *X. fastidiosa* in Europe are urgently needed to improve control efforts and to contribute towards limiting the spread of *X. fastidiosa* epidemics.

## Supplementary information

Supplementary table 1 and figure 1
