# Supplementary material for: Phenology, seasonal abundance and stage-structure of spittlebug (Hemiptera: Aphrophoridae) populations in olive groves in Italy
Source: Sci Rep. 2019 Nov 27;9:17725. doi: 10.1038/s41598-019-54279-8 (PMC6881369; doi:10.1038/s41598-019-54279-8)

## **Supplementary material**

**Phenology, seasonal abundance and stage-structure of spittlebug (Hemiptera: Aphrophoridae) populations in olive groves in Italy.**

Nicola Bodino, Vincenzo Cavalieri, Crescenza Dongiovanni, Elisa Plazio, Matteo Alessandro Saladini, Stefania Volani, Anna Simonetto, Giulio Fumarola, Michele Di Carolo, Francesco Porcelli, Gianni Gilioli, Domenico Bosco^1^

^1^Corresponding author: e-mail: [domenico.bosco@unito.it](mailto:domenico.bosco@unito.it)

CNR–Istituto per la Protezione Sostenibile delle Piante, Strada delle Cacce, 73, 10135 Torino, Italy; Dipartimento di Scienze Agrarie, Forestali e Alimentari, Università degli Studi di Torino, Largo Paolo Braccini, 2, 10095 Grugliasco, Italy

**Supplementary Table S1** characteristics of surveyed olive groves

| **Region/ Position** | | **Municipality** |  | **long** | **lat** | **elevation (m a.s.l.)** | **Olive cultivar** | **Surrounding soil use** |
| --- | --- | --- | --- | --- | --- | --- | --- | --- |
| Apulia | |  |  |  |  |  |  |  |
|  | coastal | Valenzano |  | 16.877108 | 41.05491 | 70 | Mixed | olive groves, vineyards, residentials, arable crops, fruit orchards, fallow farmlands |
|  |  |  |  |  |  |  |  |  |
|  | inland | Locorotondo 2016 |  | 17.341667 | 40.75888 | 370 | Coratina | residential, arable crops, vineyards, olive groves, broad-leaved forest |
|  | Inland | Locorotondo 2017-2018 |  | 17.285722 | 40.78675 | 366 | Coratina | olive groves, arable crops, fallow farmlands, broad-leaved forest |
|  |  |  |  |  |  |  |  |  |
| Liguria | |  |  |  |  |  |  |  |
|  | coastal | Finale Ligure |  | 8.36316574 | 44.18108 | 255 | Mixed | olive groves, vineyards, mixed woodland, Mediterranean trees/shrubs community in evolution |
|  |  |  |  |  |  |  |  |  |
|  | inland | Arnasco |  | 8.11739921 | 44.07641 | 260 | Taggiasca | olive groves, broad-leaved forest, residentials, burnt areas |

**Supplementary Figure S1** Monthly mean (± SD) temperatures (°C) measured in coastal and inland Mediterranean olive groves in Apulia and Liguria


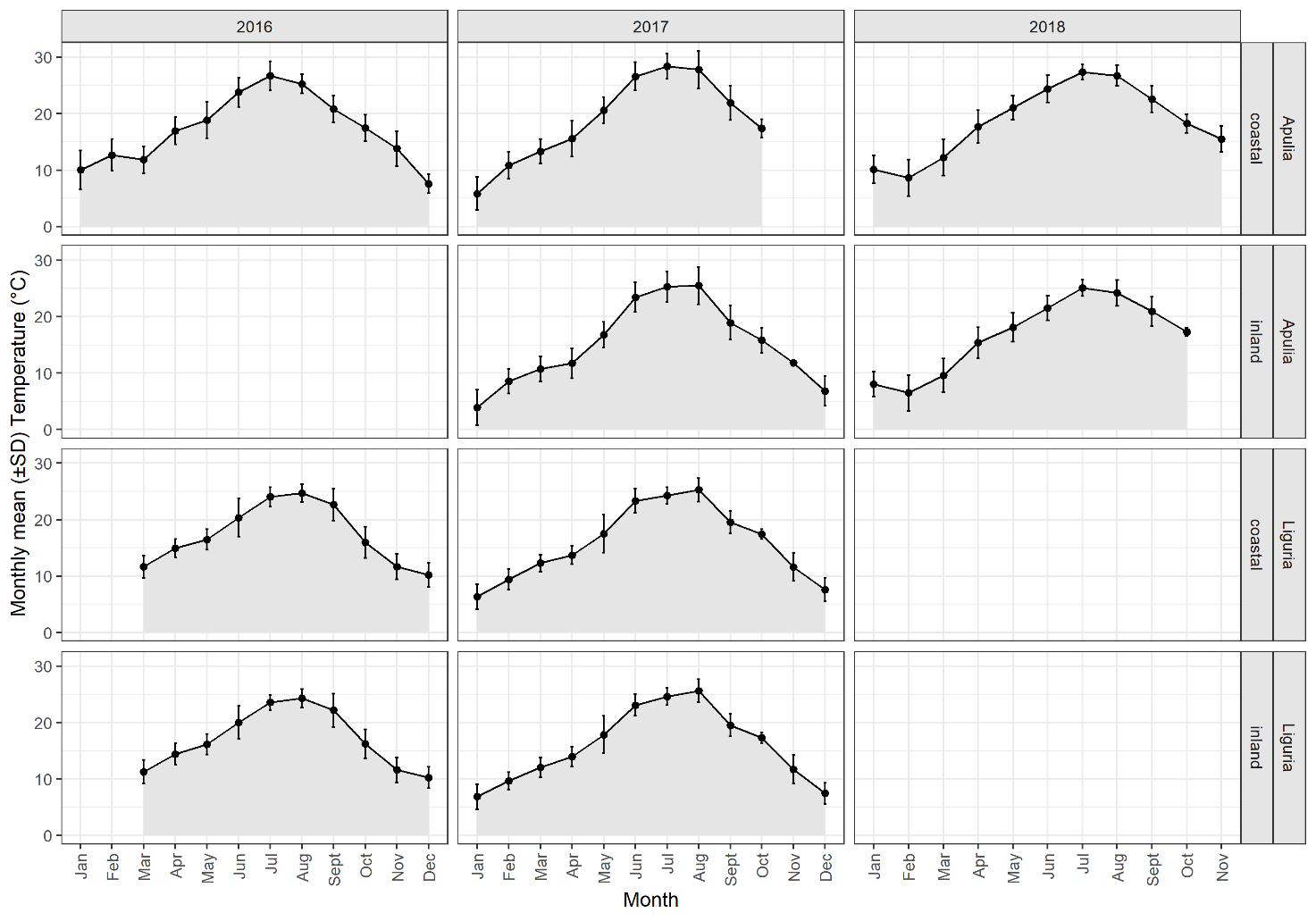

Supplement: Supplementary file 1 — Supplementary table 1 and figure 1 [file 41598_2019_54279_MOESM1_ESM.docx]
